# Supplementary material for: MEME-LaB: motif analysis in clusters
Source: Bioinformatics. 2013 May 14;29(13):1696–7. doi: 10.1093/bioinformatics/btt248 (PMC3694638; doi:10.1093/bioinformatics/btt248)
Supplement: Supplementary Data [file supp_btt248_Supplementary_Data.zip › Supplementary_Data/Results_Files/which_gene_ids/cluster9_motif1.html]

Cluster 9 Motif 1


Cluster 9 Motif 1 is found in the following promoters

| Gene ID | Strand | Position\* |
| --- | --- | --- |

|  |  |  |
| --- | --- | --- |
| AT1G01080 | - | 157/500 |
| AT1G01610 | + | 174/500 |
| AT1G01970 | + | 309/500 |
| AT1G04680 | + | 210/500 |
| AT1G05070 | + | 282/500 |
| AT1G06190 | + | 486/500 |
| AT1G07110 | + | 264/500 |
| AT1G07440 | - | 104/500 |
| AT1G08520 | + | 372/500 |
| AT1G09340 | + | 108/500 |
| AT1G10522 | - | 208/500 |
| AT1G10960 | + | 396/500 |
| AT1G11820 | - | 32/500 |
| AT1G12250 | - | 288/500 |
| AT1G13380 | + | 330/500 |
| AT1G14345 | + | 471/500 |
| AT1G15290 | - | 451/500 |
| AT1G15410 | + | 40/500 |
| AT1G16020 | + | 30/500 |
| AT1G16460 | + | 392/500 |
| AT1G16750 | - | 281/500 |
| AT1G16920 | - | 76/500 |
| AT1G19000 | - | 125/500 |
| AT1G19715 | - | 384/500 |
| AT1G20650 | + | 319/500 |
| AT1G21560 | + | 47/500 |
| AT1G21600 | + | 374/500 |
| AT1G22700 | - | 473/500 |
| AT1G28100 | - | 196/500 |
| AT1G29070 | - | 273/500 |
| AT1G29470 | - | 449/500 |
| AT1G29670 | - | 5/500 |
| AT1G31190 | - | 344/500 |
| AT1G31420 | - | 476/500 |
| AT1G32550 | - | 162/500 |
| AT1G36160 | - | 408/500 |
| AT1G41830 | - | 488/500 |
| AT1G42550 | + | 86/500 |
| AT1G44920 | - | 410/500 |
| AT1G48520 | + | 363/500 |
| AT1G49480 | - | 251/500 |
| AT1G51100 | - | 332/500 |
| AT1G51630 | + | 94/500 |
| AT1G54350 | + | 124/500 |
| AT1G54500 | - | 290/500 |
| AT1G55490 | + | 101/500 |
| AT1G56500 | + | 205/500 |
| AT1G56580 | - | 281/500 |
| AT1G59990 | + | 242/500 |
| AT1G60010 | + | 141/500 |
| AT1G60550 | + | 441/500 |
| AT1G60600 | - | 113/500 |
| AT1G60950 | + | 372/500 |
| AT1G62330 | + | 443/500 |
| AT1G62750 | + | 302/500 |
| AT1G62780 | - | 8/500 |
| AT1G63610 | - | 241/500 |
| AT1G64510 | - | 309/500 |
| AT1G64720 | + | 251/500 |
| AT1G64770 | - | 325/500 |
| AT1G65260 | - | 349/500 |
| AT1G66970 | - | 277/500 |
| AT1G67700 | - | 322/500 |
| AT1G67840 | - | 160/500 |
| AT1G68520 | + | 420/500 |
| AT1G70280 | - | 487/500 |
| AT1G70410 | + | 423/500 |
| AT1G70580 | - | 450/500 |
| AT1G71260 | - | 277/500 |
| AT1G71720 | + | 454/500 |
| AT1G72030 | - | 488/500 |
| AT1G72090 | + | 46/500 |
| AT1G72640 | - | 151/500 |
| AT1G74520 | - | 301/500 |
| AT1G74920 | + | 288/500 |
| AT1G74970 | - | 347/500 |
| AT1G75350 | + | 231/500 |
| AT1G75380 | + | 335/500 |
| AT1G77090 | - | 361/500 |
| AT1G78010 | - | 338/500 |
| AT1G79510 | + | 459/500 |
| AT1G79720 | + | 77/500 |
| AT2G01760 | - | 153/500 |
| AT2G02740 | + | 60/500 |
| AT2G03120 | + | 158/500 |
| AT2G03350 | + | 244/500 |
| AT2G04039 | - | 256/500 |
| AT2G04790 | - | 31/500 |
| AT2G05920 | - | 453/500 |
| AT2G07340 | + | 285/500 |
| AT2G17440 | - | 355/500 |
| AT2G18290 | - | 231/500 |
| AT2G19360 | + | 220/500 |
| AT2G23390 | - | 257/500 |
| AT2G24280 | + | 337/500 |
| AT2G25840 | - | 422/500 |
| AT2G26840 | - | 392/500 |
| AT2G28000 | - | 112/500 |
| AT2G28800 | - | 126/500 |
| AT2G28950 | + | 435/500 |
| AT2G29180 | + | 471/500 |
| AT2G30390 | + | 70/500 |
| AT2G30695 | + | 301/500 |
| AT2G31840 | - | 8/500 |
| AT2G32180 | + | 298/500 |
| AT2G32470 | - | 239/500 |
| AT2G33530 | + | 194/500 |
| AT2G33990 | - | 111/500 |
| AT2G34310 | + | 372/500 |
| AT2G34680 | - | 482/500 |
| AT2G35130 | - | 79/500 |
| AT2G36230 | - | 356/500 |
| AT2G38120 | + | 474/500 |
| AT2G39190 | - | 402/500 |
| AT2G40280 | + | 409/500 |
| AT2G40760 | + | 349/500 |
| AT2G42600 | + | 445/500 |
| AT2G42730 | + | 331/500 |
| AT2G44210 | + | 440/500 |
| AT2G45470 | - | 297/500 |
| AT2G45590 | + | 198/500 |
| AT2G45820 | + | 288/500 |
| AT2G47940 | + | 155/500 |
| AT3G01480 | - | 222/500 |
| AT3G01660 | - | 199/500 |
| AT3G02660 | - | 137/500 |
| AT3G02690 | - | 230/500 |
| AT3G04940 | + | 55/500 |
| AT3G05410 | - | 179/500 |
| AT3G05910 | + | 91/500 |
| AT3G06145 | - | 384/500 |
| AT3G06430 | - | 150/500 |
| AT3G06510 | + | 150/500 |
| AT3G06980 | - | 448/500 |
| AT3G07310 | + | 394/500 |
| AT3G08740 | - | 68/500 |
| AT3G09650 | - | 480/500 |
| AT3G10270 | + | 120/500 |
| AT3G10840 | - | 361/500 |
| AT3G13120 | + | 300/500 |
| AT3G13360 | - | 454/500 |
| AT3G13560 | - | 373/500 |
| AT3G14240 | - | 478/500 |
| AT3G15480 | + | 113/500 |
| AT3G15850 | + | 148/500 |
| AT3G15970 | + | 101/500 |
| AT3G16000 | + | 463/500 |
| AT3G17040 | + | 175/500 |
| AT3G19800 | + | 230/500 |
| AT3G19820 | - | 221/500 |
| AT3G21190 | + | 431/500 |
| AT3G22420 | - | 36/500 |
| AT3G23760 | + | 7/500 |
| AT3G24150 | + | 392/500 |
| AT3G25690 | - | 166/500 |
| AT3G25920 | + | 411/500 |
| AT3G26070 | - | 453/500 |
| AT3G26300 | + | 225/500 |
| AT3G27180 | + | 308/500 |
| AT3G27230 | + | 47/500 |
| AT3G28460 | - | 322/500 |
| AT3G29290 | - | 216/500 |
| AT3G43540 | - | 327/500 |
| AT3G43800 | - | 76/500 |
| AT3G44890 | + | 409/500 |
| AT3G45860 | - | 83/500 |
| AT3G47450 | - | 294/500 |
| AT3G47650 | - | 221/500 |
| AT3G48730 | + | 234/500 |
| AT3G50685 | + | 261/500 |
| AT3G52430 | - | 14/500 |
| AT3G52750 | - | 408/500 |
| AT3G52840 | + | 252/500 |
| AT3G54090 | - | 348/500 |
| AT3G56010 | - | 387/500 |
| AT3G56650 | - | 211/500 |
| AT3G57190 | + | 418/500 |
| AT3G59040 | - | 305/500 |
| AT3G61080 | - | 376/500 |
| AT3G61220 | + | 305/500 |
| AT3G62910 | + | 137/500 |
| AT3G63190 | - | 75/500 |
| AT3G63490 | + | 326/500 |
| AT3G63520 | - | 386/500 |
| AT4G03280 | + | 188/500 |
| AT4G05090 | + | 454/500 |
| AT4G05250 | - | 447/500 |
| AT4G08685 | + | 86/500 |
| AT4G09650 | - | 476/500 |
| AT4G10120 | + | 286/500 |
| AT4G10300 | + | 205/500 |
| AT4G12830 | + | 192/500 |
| AT4G15545 | + | 201/500 |
| AT4G17810 | + | 296/500 |
| AT4G18070 | - | 297/500 |
| AT4G19100 | - | 375/500 |
| AT4G20130 | - | 487/500 |
| AT4G24280 | - | 238/500 |
| AT4G24750 | + | 334/500 |
| AT4G27900 | + | 453/500 |
| AT4G28660 | + | 122/500 |
| AT4G30620 | + | 147/500 |
| AT4G30720 | - | 254/500 |
| AT4G30950 | + | 453/500 |
| AT4G32330 | - | 480/500 |
| AT4G32980 | + | 280/500 |
| AT4G33030 | - | 198/500 |
| AT4G33220 | - | 51/500 |
| AT4G33470 | - | 487/500 |
| AT4G34260 | - | 203/500 |
| AT4G34830 | + | 119/500 |
| AT4G35020 | - | 176/500 |
| AT4G36520 | - | 136/500 |
| AT4G36530 | + | 282/500 |
| AT4G39620 | + | 478/500 |
| AT4G39710 | - | 50/500 |
| AT5G03430 | - | 405/500 |
| AT5G03650 | + | 263/500 |
| AT5G04810 | - | 151/500 |
| AT5G06340 | - | 417/500 |
| AT5G07240 | + | 47/500 |
| AT5G07580 | + | 102/500 |
| AT5G07690 | - | 386/500 |
| AT5G08050 | + | 265/500 |
| AT5G08370 | - | 277/500 |
| AT5G08470 | - | 235/500 |
| AT5G08540 | - | 385/500 |
| AT5G09320 | + | 265/500 |
| AT5G09870 | - | 111/500 |
| AT5G10560 | + | 384/500 |
| AT5G10920 | + | 120/500 |
| AT5G11330 | + | 229/500 |
| AT5G12150 | - | 218/500 |
| AT5G12250 | - | 64/500 |
| AT5G13510 | - | 133/500 |
| AT5G13650 | - | 483/500 |
| AT5G14450 | - | 380/500 |
| AT5G16000 | + | 487/500 |
| AT5G16715 | - | 416/500 |
| AT5G17170 | + | 431/500 |
| AT5G17230 | + | 267/500 |
| AT5G18570 | - | 302/500 |
| AT5G20140 | + | 422/500 |
| AT5G21222 | - | 368/500 |
| AT5G21920 | + | 197/500 |
| AT5G22340 | + | 366/500 |
| AT5G22800 | + | 226/500 |
| AT5G23280 | + | 352/500 |
| AT5G23570 | + | 378/500 |
| AT5G24314 | + | 417/500 |
| AT5G26780 | + | 288/500 |
| AT5G26850 | + | 195/500 |
| AT5G27290 | + | 89/500 |
| AT5G27390 | + | 429/500 |
| AT5G27560 | + | 127/500 |
| AT5G30510 | - | 124/500 |
| AT5G35630 | - | 300/500 |
| AT5G40380 | + | 436/500 |
| AT5G41050 | - | 349/500 |
| AT5G42070 | + | 446/500 |
| AT5G42240 | + | 453/500 |
| AT5G43310 | + | 292/500 |
| AT5G43750 | - | 163/500 |
| AT5G44520 | + | 453/500 |
| AT5G44650 | + | 433/500 |
| AT5G46580 | - | 149/500 |
| AT5G47110 | + | 399/500 |
| AT5G47190 | + | 50/500 |
| AT5G47640 | + | 127/500 |
| AT5G48545 | - | 16/500 |
| AT5G50420 | - | 411/500 |
| AT5G51010 | - | 480/500 |
| AT5G51110 | - | 206/500 |
| AT5G53170 | + | 437/500 |
| AT5G53420 | + | 101/500 |
| AT5G53500 | + | 427/500 |
| AT5G53860 | + | 273/500 |
| AT5G54600 | - | 345/500 |
| AT5G55220 | - | 266/500 |
| AT5G57040 | - | 425/500 |
| AT5G57170 | + | 279/500 |
| AT5G58480 | - | 459/500 |
| AT5G60400 | - | 265/500 |
| AT5G61340 | + | 300/500 |
| AT5G62470 | + | 389/500 |
| AT5G62840 | - | 251/500 |
| AT5G63020 | - | 443/500 |
| AT5G63530 | + | 44/500 |
| AT5G64460 | + | 470/500 |
| AT5G65810 | + | 348/500 |
| AT5G66190 | + | 129/500 |
| AT5G66530 | + | 19/500 |

**\*** Overlapping sequences are merged to avoid redundancy. As a result some sequences in the final set may be longer than the specified maximum.

Distribution

+

-

Position values represent number of bases from the 5 prime end of the sequence provided to MEME and the length of that sequence. It is these figures expressed as proportions that are used for statistical analysis.

Probability value of comparison of these values to a linear distribution, p = 0.0023

The motif is found 149 times on the + stand and 143 times on the - strand.

Probability value that it is evenly distributed between the two strands, p = 0.38

Move the mouse pointer over the gene ids to highlight the corresponding marker
